# Integrating AI and RNA biomarkers in cancer: advances in diagnostics and targeted therapies

**DOI:** 10.1186/s12964-025-02434-2

**Published:** 2025-10-10

**Authors:** Bashdar Mahmud Hussen, Snur Rasool Abdullah, Hazha Jamal Hidayat, Majid Samsami, Mohammad Taheri

**Affiliations:** 1 Department of Clinical Analysis, College of Pharmacy, Hawler Medical University, Erbil, Kurdistan Region Iraq; 2 Department of Medical Laboratory Science, College of Health Sciences, Lebanese French University, Erbil, Kurdistan Region Iraq; 3 Department of Biology, College of Education, Salahaddin University- Erbil, Erbil, Kurdistan Region Iraq; 4 Phytochemistry Research Center, Shahid Beheshti University of Medical Sciences, Tehran, Iran; 5 Institute of Human Genetics, Jena University Hospital, Jena, Germany; 6 Research Institute for Urology and Nephrology, Shahid Beheshti University of Medical Sciences, Tehran, Iran

**Keywords:** Artificial intelligence (AI), RNA biomarkers, Cancer, Therapeutic target

## Abstract

Early detection and personalized treatment strategies are essential for enhancing patient outcomes, as cancer continues to be a significant cause of mortality on a global basis. In clinical practice, the identification and validation of reliable biomarkers for cancer diagnosis, prognosis, and therapeutic monitoring continue to present significant challenges. The present study explores the current state and applications of artificial intelligence-driven approaches in the identification and usage of RNA biomarkers for cancer diagnostics and therapeutics. In various aspects of cancer management, we explore the integration of machine learning and deep learning algorithms with a variety of RNA biomarker classes, such as circRNAs, miRNAs, and lncRNAs. Improved detection, subtype categorization, prognosis prediction, and treatment response monitoring are all possible due to AI-powered approaches that can efficiently analyse complex RNA expression patterns, discover novel biomarkers, and explain their functions in cancer biology. There are still many obstacles to overcome in the biomarker development, validation, and clinical application processes, despite the fact that RNA biomarkers hold great potential to transform cancer treatment by improving early detection and individualized therapy methods. Integrating AI with RNA biomarker research is a crucial strategy with enormous promise for precision oncology and better patient care all the way through the cancer spectrum, from risk prediction to recurrence management.

## Introduction

The term “biomarkers” refers to medical signs or detectable changes in biological substances that are associated with healthy or diseased situations [1]. Over the years, clinical trials have utilized a wide range of biomarkers to guide disease diagnosis and prognosis [2]. Typically, biomarkers in cancer care have three forms of healthcare utility: diagnostic, prognostic, and predictive.

The significance of RNAs in regulating many biological processes has led to their recognition as minimally invasive biomarkers for disease diagnosis and prognosis that are considered effective [3–7]. An abundance of efficient RNA-detection techniques has emerged recently due to the rapid expansion of RNA biomarker studies [8]. The majority of these technologies are based on molecular biology methods, the most common of which are RNA sequencing, microarrays, and RT-qPCR. At the transcriptome level, high-throughput sequencing techniques have enabled the detection of mRNAs (RNAs that code for proteins) and many non-coding RNAs, such as miRNA, short nucleolar RNA, and small nuclear RNA in biological samples.

mRNA is the most studied form of RNA used as a biomarker. As a biomarker for clinical outcome, multi-gene expression patterns have been exploited in numerous cancer research [9]. Recent studies have demonstrated the existence of additional non-protein RNAs with significant functional roles beyond mRNAs. Many of them, including miRNAs, circRNAs, lncRNAs, and others, have the potential to be biomarkers besides their additional uses [10]. However, the extensive and complex datasets produced by high-throughput RNA sequencing and omics technologies necessitate sophisticated analytical tools to discern significant patterns. Artificial Intelligence (AI) is a disruptive force revolutionizing cancer diagnoses and therapies by deciphering the concealed signatures within RNA biomarker data.

AI, especially machine learning and deep learning, has transformed the analysis of intricate biological data, facilitating the detection of nuanced yet clinically significant RNA expression patterns that conventional statistical techniques fail to capture [11, 12]. By utilizing AI, researchers may examine extensive transcriptome databases to identify new RNA biomarkers, forecast illness development, and enhance treatment techniques [13]. For instance, algorithms powered by AI have been utilized to categorize cancer subtypes according to miRNA expression profiles, enhancing diagnostic precision beyond traditional histopathological techniques [14]. Similarly, predictive models employing lncRNA signatures have demonstrated considerable effectiveness in forecasting patient outcomes and treatment responses, hence facilitating personalized intervention strategies [15, 16].

In cancer diagnostics, AI improves the identification of RNA biomarkers from liquid biopsies, offering a non-invasive and extremely sensitive method for early cancer screening. In BC, support vector machines (SVMs) and neural networks have been trained using circulating RNA data to differentiate between benign and malignant diseases accurately [17, 18]. Furthermore, AI-driven technologies amalgamate multi-omics data-integrating RNA sequencing with genomic and proteomic profiles, to produce complete diagnostic signatures that enhance early detection rates and minimize false positives [19].

In pharmaceuticals, AI is crucial for locating RNA-based targets in precision oncology. For example, reinforcement learning models have been employed to forecast tumor responses to RNA-interference therapies or immunomodulatory treatments, hence optimizing therapy selection for individual patients [20, 21]. Additionally, AI enhances the design of RNA treatments, including small interfering RNAs (siRNAs) and antisense oligonucleotides (ASOs), by forecasting their binding affinities and off-target effects, thus expediting drug development processes [22].

Despite these advancements, challenges persist, including the need for robust validation in diverse clinical cohorts, addressing biases in training datasets, and ensuring the interpretability of AI models for clinical adoption. Ethical considerations, such as data privacy and algorithmic transparency, must also be addressed to foster trust in AI-driven healthcare solutions. However, this study shows the interactive integration of AI and RNA biomarkers in cancer diagnostics and therapies, emphasizing advanced methodology, successful implementations, and prospective developments.

## RNA biomarkers and AI in cancers

RNAs are transcriptionally and post-transcriptionally regulated in addition to being genetic information transmitters [23]. Despite RNAs’ instability in acidic environments, they are readily detectable and quantifiable at extremely low concentrations [24]. The sensitivity and specificity of RNA biomarkers are higher than those of protein biomarkers. Furthermore, due to the need for a specialized antibody for protein detection, the cost of RNA biomarkers is substantially lower than that of protein biomarkers. Further, it is possible to measure the amounts of RNA expression across the entire genome by using next-generation sequencing. Novel transcripts, including lowly expressed non-coding RNAs (ncRNAs), and fine-grained changes in expression can be more accurately detected as RNA sequencing depth increases [25].

There would be a positive or negative correlation between disease pathology and the differential expression of particular genes. Patterns of multi-gene expression have been utilized as a biomarker for therapeutic success in numerous cancer investigations. As an example, the 50-gene panel known as PAM50 has been effectively used for breast cancer (BC) classification [26]. Similarly, BRCA1 and BRCA2 high-penetrance mutations in ovarian and breast carcinoma are well known for their powerful effects and substantial association with the disease. As a result, mutations in these two genes are excellent candidates for use as biomarkers for cancer risk assessment; they correspond to syndromes that predispose to cancer and hereditary malignancies [27].

On the other hand, there have been many recent discoveries of functionally relevant RNAs other than mRNAs that do not code for proteins and a number of them have potential as biomarkers. For instance, miRNAs, circRNAs, and lncRNAs are involved in transcription and post-transcriptional control. While some ncRNAs act as oncogenes or tumor suppressors, others perform essential functions in cellular differentiation, proliferation, and the apoptosis process [28].

The integrated analysis of ML approaches and differentially expressed genes (DEGs) has further highlighted the significance of specific genes in cancer pathogenesis. In lung cancer (LC) samples, the top five upregulated genes identified were COL11A1, TOP2A, SULF1, DIO2, and MIR196A2, whereas the top five downregulated genes were PDK4, FOSB, FLYWCH1, CYB5D2, and MIR328. Associated genes implicated in relevant pathways or co-expression networks were also identified [29]. Among the ML algorithms employed, Random Forest and XGBoost were particularly effective in identifying common genes, underscoring their potential role in LC pathogenesis. The MLP algorithm achieved the highest accuracy in classifying samples based on the complete gene set. Furthermore, protein-protein interaction analysis revealed 10 hub genes pivotal in LC pathogenesis: COL1A1, SOX2, SPP1, THBS2, POSTN, COL5A1, COL11A1, TIMP1, TOP2A, and PKP1 [29]. These findings exemplify how integrating RNA biomarker profiling with advanced computational approaches can offer solid conclusions about cancer biology.

Apart from RNAs present in tumor cells, recent studies have identified a number of exRNAs. ExRNAs include a wide variety of RNA types, including miRNA, siRNA, piRNA, snoRNA, tRNA, circRNA, and lncRNA. They are also present in a wide variety of other biological fluids, such as blood, saliva, bile, urine, breast milk, and cerebrospinal fluid [30–33]. Moreover, Hanahan and Weinberg’s definitions of RNA biomarkers suggested that these molecules may contribute significantly to some of the cancer hallmarks, including maintaining proliferative signaling, evading growth inhibitors, surviving apoptosis, facilitating replicative immortality, and triggering angiogenesis, invasion, and metastasis [34].

Additionally, a large number of biomarker databases focus on certain diseases. As an example, the Human miRNA Disease Database (HMDD) is an experimentally supported database of human miRNA-disease relationships that includes information from genetics, epigenetics, circulating miRNAs, and interactions between miRNAs and their targets. The Colon Rectal Cancer Gene (CoReCG) database contains information on genes and pathways that are actually implicated in colorectal carcinoma. It also includes genes that are altered, polymorphic, or differently expressed at different stages of the cancer [35]. The Osteosarcoma database is a platform for evaluating miRNAs and protein-coding genes linked to osteosarcoma. It is based on manual annotation and results from literature searches. Potentially used as biomarkers for this disease [36].

Despite the previously mentioned disease-specific databases, there are additional biomarker databases that provide more comprehensive and complete data. As an example, MIRUMIR incorporates miRNA datasets that are publicly available and have been annotated with survival statistics for patients. One potential use of MIRUMIR is to assess the predictive power of specific microRNAs for cancer patients’ survival rates [37]. The National Cancer Institute (NCI) in the US built the Biomarker Database (BMDB). It is part of the Early Detection Research Network (EDRN). Additionally, a number of databases have been created with the express purpose of storing biomarkers that are located outside of cells, or in exosomes. Example: exRNA Atlas compiles the most recent data from several exRNA studies, such as exRNA profiling information gleaned from RT-qPCR and short RNA sequencing, standardized exRNA methods, and an abundance of other helpful technologies and tools [38]. Using miRandola, a database of projected extracellular miRNA biomarkers, researchers might investigate the cellular function of miRNAs outside of cells [39]. Further, with the intention of enhancing our comprehension of exosomal proteins, RNAs, and lipids, ExoCarta collects information gathered from many legal and unlicensed research sources [40, 41].

Clinical oncology research currently focusses on understanding the genetic roots of cancer by identifying the detailed biological framework of neoplastic cell proliferation. Further, it aimed to process the millions of relevant cases in biological computation and big data in order to address the present situation of the increasing global cancer-related deaths [42].

‘AI’ is a general term used to describe functions that are often performed by humans. Learning algorithms to generate predictions based on past experiences is known as machine learning (ML), a subfield of AI. ML can be classified into two primary categories of learning: supervised learning, in which the computer is provided access to result data, and unsupervised learning, in which no such data is given. AI-powered clinical decision-making has the potential to enhance the use of high-resolution imaging and NGS for early diagnosis and prediction [43, 44].

**Fig. 1:**
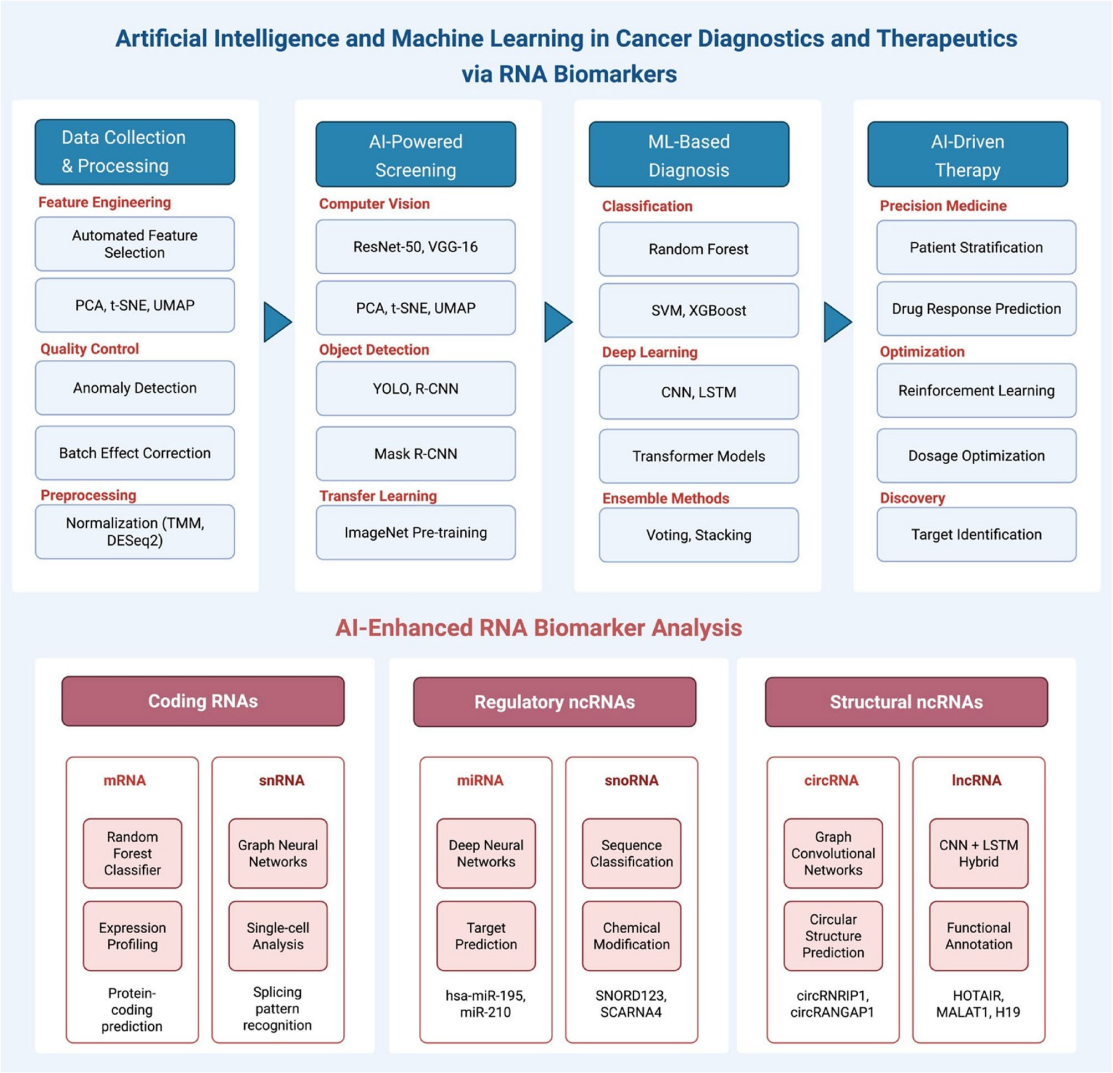
An AI/ML Framework for RNA biomarkers-based cancer diagnostics and therapeutics shows how AI/ML models can be applied to four critical steps in cancer care. Feature engineering algorithms (PCA, t-SNE, UMAP), anomaly detection for quality control, and normalisation methods (TMM, DESeq2) for preprocessing are all part of data collection and processing. By combining object detection methods (YOLO, R-CNN), transfer learning from ImageNet, and computer vision models (ResNet-50, VGG-16, and U-Net segmentation), AI-Powered Screening achieves a high sensitivity level in cancer detection. To attain an area under the curve (AUC) for diagnostic accuracy, ML-based diagnosis combines ensemble methods (voting, stacking), deep learning architectures (CNN, LSTM, Transformers), and classification algorithms (Random Forest, SVM, XGBoost). Through patient stratification, dosage optimization, target identification, and drug response prediction via reinforcement learning, AI-driven therapy applies precision medicine. AI is demonstrated in the RNA biomarker panel for both coding and non-coding RNAs, with mRNA and snRNA processed by Random Forest and Graph Neural Networks, respectively, and miRNA and circRNA by Graph Convolutional Networks and CNN + LSTM hybrids, respectively

Developing large data sets and using specialist bioinformatic tools would also lead to the delivery of potential therapies, the creation of unique specific drugs, and the development of novel biomarkers for oncological diagnosis. Moreover, it is thought that incorporating AI into clinical decision-making will improve the probability of early disease prediction and diagnosis through the use of NGS and high-resolution imaging methods [44]. Additionally, the generation of extensive datasets and employing specialized bioinformatic tools may lead to the emergence of innovative biomarkers for cancer detection, the design of new personalized medications, and the provision of prospective treatment protocols [45] (Fig. 1).

The complete application of AI for medical diagnostics is still in its early stages. Nonetheless, more information is becoming available for the use of AI in the diagnosis of various illnesses, including cancer. For example, there was a dramatic shift in the domain of cancer diagnostics with the introduction of an AI-powered BC detection system [18]. The FDA has authorized QuantX, the first computer-aided system, for the identification of BC. QuantX gives radiologists more clinical patient-related data needed for diagnosis and has a 20% improvement in accuracy compared to competing imaging technologies [42]. On top of that, McKinney et al. demonstrated that their AI system is superior to human specialists when it comes to BC prediction [46]. They carefully selected a large representative dataset from the UK and a sizable, enhanced dataset from the USA. There was a 5.7% and 1.2% absolute decrease in false positives and a 9.4% and 2.7% absolute decrease in false negatives (USA and UK). They offered proof that the technique could be applied from Britain to the United States of America. Therefore, this thorough evaluation of the AI system enables the accurate and efficient detection of BC through clinical trials. Similarly, the AI program that was developed using massive amounts of mammography data performed better than radiologists when it succeeded in detecting BC. For instance, AI-driven diagnosis exhibited a sensitivity of 90%, surpassing the 78% sensitivity of radiologists when it determined mass-based BC diagnoses. Moreover, compared to radiologists, AI was 91% more accurate in diagnosing early BC [47].

Further, the AI system showed promise in a number of areas, including skin cancer prediction, primary treatment choice suggestion, multi-class disorder rendering among 134 disorders, and medical staff efficiency. At 0.928 ± 0.002, the area under the curves for detecting malignancy was determined [48]. In addition to speeding up processes and improving accuracy, AI could reduce the likelihood of human errors. By giving doctors access to information and help in real-time, AI has the potential to improve medical decisions soon.

Clinical laboratory tests provide important information for disease diagnosis, treatment, and monitoring. Modern healthcare is constantly integrating new technologies to support clinical decision-making and patient safety [49]. By enhancing the precision, speed, and efficacy of laboratory procedures, AI may revolutionize clinical tests in laboratories. AI function in clinical laboratories is rapidly developing and growing. In order to detect, identify, and quantify genes; diagnose and classify cancer diseases; and forecast clinical outcomes, various ML algorithms were developed [50, 51]. Biological genomic data, gene sequencing, original specimen metagenomic sequencing results, microscopic imaging, and other data sources were utilized by these ML systems to construct the AI diagnostic [51].

With 85% of cases occurring in low- and middle-income nations, cervical cancer ranks as the fourth most frequent malignancy in women [52]. When it comes to manually evaluating Pap smear slide samples, some nations have less than one pathologist per million, which puts cancer diagnosis capabilities at a critical level [53]. Results showed that the AI system is very sensitive (96–100% sensitivity) in identifying abnormal cells in cervical smears, with sensitivity increasing from 82 to 93% in low-grade lesions compared to low-grade lesions (82–86%) in research including 740 patients belonging to a rural clinic in Kenya [54]. Atypia of low grade was detected in 5% of samples and high grade in 8% of samples, according to the algorithm. In addition to these results, the algorithm did not incorrectly recognize any samples that were found to be negative by an expert pathologist.

The field of histology has identified AI as the third revolutionary change [55]. Despite its extensive use in Haematoxylin and Eosin stained slides for the detection of image-based cancer biomarkers, AI-based feature extraction is rarely utilized in multiplexed fluorescence immunohistochemistry due to some challenges [56]. For example, in multiplexed fluorescence immunohistochemistry, in particular, there are a number of obstacles to its application. This technique is quite effective, but it generates multichannel images that are extremely multiplexed, demanding sophisticated algorithms for feature extraction. It takes a lot of processing power and complex models to deal with this kind of data.

### AI-based models for RNA biomarker discovery

**Fig. 2:**
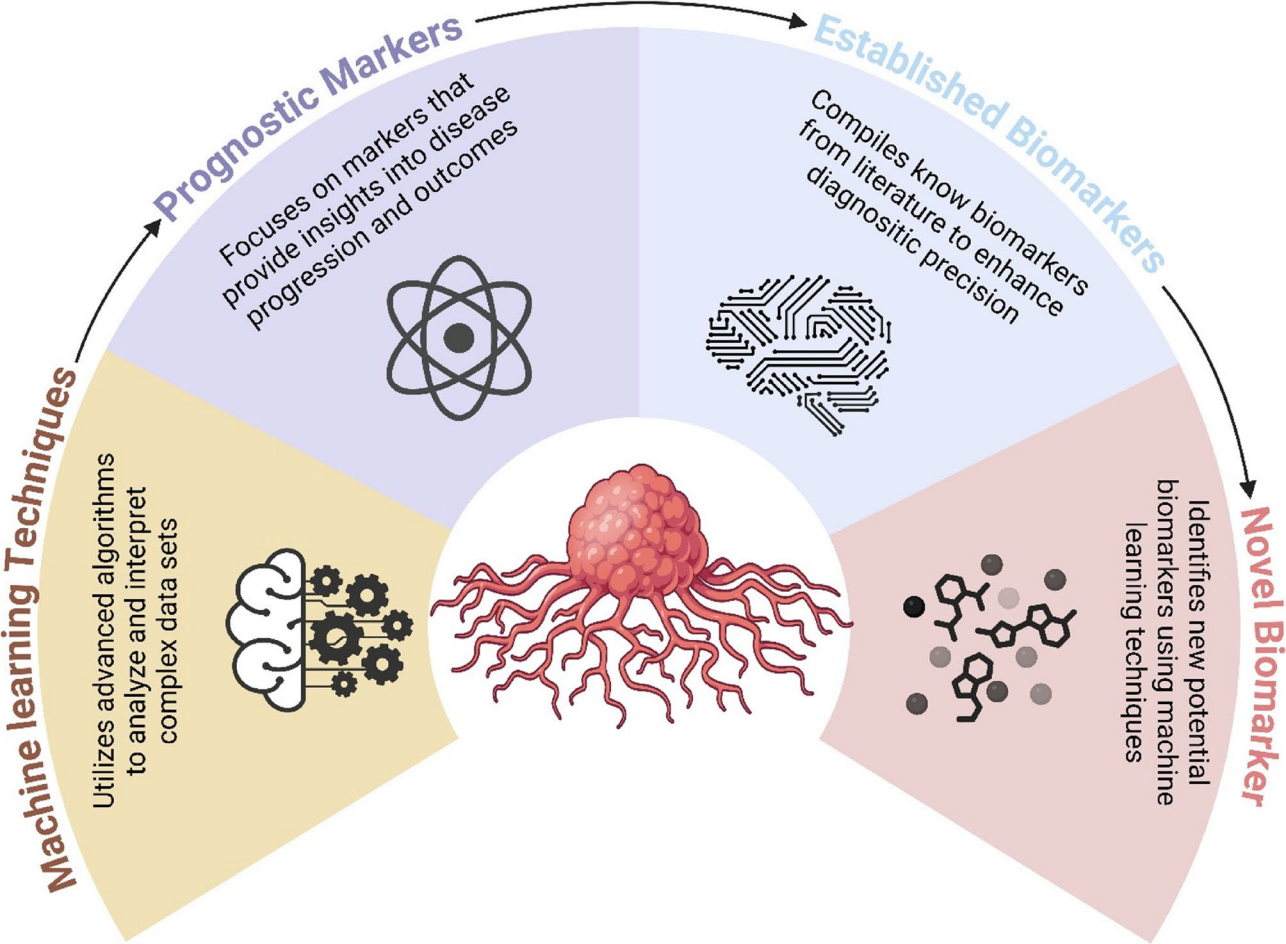
The schematic diagram represents the utilization of AI algorithms to discover and analyze novel biomarkers. The schematic diagram was created using BioRender.com

AI has started to be used as a computer tool to identify and evaluate putative cancer biomarkers (Fig. 2). Typically, DL, ML, and computational search methods comprise AI techniques related to biomarker identification [57]. Structured, sequential approaches are used by computational search algorithms to assess a set of variables, but machine learning techniques have an innate feedback loop that allows model parameters to be changed during training and then validated during testing. These methods frequently make use of controlled learning, which is a method that depends on outcome data and labeled variables.

Numerous investigations have used machine learning to evaluate RNA-seq data in order to find novel transcript biomarkers that distinguish between tumor and normal tissues. Gupta et al. used this method to identify three novel biomarkers that may distinguish between HCC tumor and normal liver tissue: SPON2-203, PARP2-202, and CYREN-211 [58]. The last transcript was found to be non-coding, whereas the first two were found to be protein-coding. This shows that a transcript’s coding status is not always a reliable indicator of its biomarker significance, and non-coding transcripts can nevertheless play important regulatory roles. The greatest predictive value was also found to be held by the established biomarkers for HCC that they had compiled from a thorough literature study (Gupta et al. Table 1), but the addition of the transcripts above enhanced the overall precision.

**Table 1:** Shows the AI-driven discovery and application of different RNAs in cancer diagnosis

**RNA biomarker**	**Kind of RNA**	**Disease application**	**Data sources**	**Applied AI techniques**	**Evaluation metrics**	**Challenges addressed**	**Clinical impact**	**Ref.**
mRNA and lncRNA	2 mRNA (S100A9 and S100A7) and 2 lncRNA (HSALNG0002446 and HSALNG0129303) biomarkers	BC, psoriasis, and leukemia.	RNA-seq and microarray, Gene annotation, Co-expression network analysis, Gene Ontology	BAMBI, BioDiscML, ILRC, and ECMarker techniques	BAMBI Accuracy = 95.20%	Poor reproducibility on independent datasets, an inability to process omics data directly, difficulty identifying noncoding RNAs as biomarkers, and a lack of biological interpretation and clinical utility are other problems.	Early diagnosis, monitoring, and prognosis,	[59]
					BioDiscML Accuracy = 91.12%			
					ILRC Accuracy = 90.40%			
					ECMarker Accuracy = 71.68%			
mRNA, miRNA	mRNAs (NPNT, CDK18, KIF5A, SPACA6, TCEA3, SYTL1, ARRDC2, APLN) and hsa-miR-423-3p, hsa-miR-33a, hsa-miR-640, hsa-miR-362-3p, hsa-miR-491-5p, hsa-miR-206, hsa-miR-548b-3p, hsa-miR-127-3p, hsa-miR-106a_hsa-miR-17, hsa-miR-424, hsa-miR-577, hsa-miR-873, hsa-miR-651, hsa-miR-199b-5p, hsa-miR-192, hsa-miR-199a-5p, hsv1-miR-H1	Breast cancer	multi-omics data	MOGONET	-	Allowing patient classification and biomarker identification.	Early diagnosis	[60]
mRNA	50 mRNAs	BC	TCGA and METABRIC databases	Ensemble models such as TCGA and METABRIC	Accuracy = 82.55%	Limited sample size	Early diagnostic panel	[61]
					Sensitivity = 84.55%			
					Specificity = 79.22%			
miRNA	hsa-miR-195 + hsa-miR-210 + hsa-miR-503; hsa-miR-210 + hsa-miR-375 + hsa-miR-503 and hsa-miR-210 + hsa-miR-483-5p + hsa-miR-503	ACA	RT-qPCR, Clinical databases	R packages nnet and caret)	Sensitivity = 90%	A limited set of miRNAs was examined, and the sizes of the cohorts and FFPE samples were used.	Early diagnosis	[62]
					Specificity = 90%			
	hsa‑miR‑10b‑5p, hsa‑miR‑183‑5p,	HCC	RT-qPCR, Clinical databases	GSE63046	AUC = 98.2, 97	Small sample size, uncleared molecular mechanism, and minimally invasive techniques for diagnosis.	Early diagnosis and prediction of prognosis	[63]
	hsa‑miR‑10b‑3p,							
	hsa‑miR‑182‑5p, hsa‑miR‑224‑5p							
	hsa-miR-362-3p, hsa-let-7i-3p, hsa-miR-3651	15 cancer types	TCGA database	Cancer*Sig*	Accuracy = 84.27%.	Utilizing the TCGA data alone.	Early identification of cancer stage and development of targeted therapy	[64]
					Sensitivity = 81%			
					Specificity = 80%			
					AUC = 0.80 ± 0.06			
	hsa-miR-5100, hsa-miR-1233-5p, hsa-miR-6800-5p, hsa-miR-4783-3p, hsa-miR-4532, hsa-miR-4787-3p, hsa-miR-1290, hsa-miR-1228-5p, hsa-miR-3184-5p, hsa-miR-320b	OC	GSE106817 dataset	LR (statistical), DT and RF (tree-based), ANN and XGB (machine learning)	LR Accuracy = 100%	Small sample sizes and pathological information were not available in the GSE106817 dataset.	Early diagnosis and prediction of prognosis	[65]
					LR Sensitivity = 100%			
					LR Specificity = 100%			
					DT Accuracy = 91.30%			
					-DT Sensitivity = 92.50%			
					DT Specificity = 90.38%			
					RF Accuracy = 97.83%.			
					RF Sensitivity = 95%			
					RF Specificity = 100%			
					ANN Accuracy = 100%			
					ANN Sensitivity = 100%			
					ANN Specificity = 100%			
					XGB Accuracy = 98.91%			
					XGB Sensitivity = 97.50%			
					XGB Specificity = 100%			
					AUC = 100%			
	miRNA-10b-5, miRNA-186-5p, miRNA-30e-5p, miRNA-30e, miRNA-21, miRNA-21, miRNA-30e	CRC	Microarray, RNA-seq	GSE41012 and GSE54088	-	Insufficient robustness resulting from the variability of various populations	Early diagnosis and development of targeted therapy	[66]
	miR-1914, miR-149, miR-203, miR-135a-2, miR-9–1	OC	Clinical databases	ANOVA feature selection method	Accuracy = 73%%	Small sample size	Early diagnosis and prediction of prognosis	[67]
					AUC = 0.65			
lncRNA	RP11-598F7.5, FOXD2-AS1,	STAD	qRT-PCR and GSE27342 dataset	Decision tree model, random forests model, SVM	Decision tree model Sensitivity = 97.1%	Optimal diagnostic lncRNAs for STAD were found in small sample numbers, although their biological activities remain unexamined.	Preliminary diagnostic biomarkers of STAD. LINC01235 served as a prognostic biomarker.	[68]
	LINC01235				Decision tree model Specificity = 75.0%			
					AUC = 0.797			
					Random forests			
					model Sensitivity = 96%			
					Random forests model Specificity = 96.9%			
					AUC = 0.981			
					SVM Sensitivity = 97.1%			
					SVM Specificity = 96.9%			
					AUC = 0.983			
	Twenty-seven stable recurrence-associated lncRNAs were discovered from multicenter cohorts.	CRC	GEO database, qRT-PCR	RSF, Enet, Lasso, Ridge, stepwise Cox, CoxBoost, plsRcox, SuperPC, GBM, survival- SVM	AUCs = 0.755	The study’s retrospective samples revealed inadequate clinical and molecular traits on public datasets, and the functions of most lncRNAs from CMDLncS in stage II/III CRC remain ambiguous.	Development of targeted therapy	[69]
	Multiple lncRNAs	BC	RNA-sequencing	RDeepNet, RFS	AUC = 0.98, AUC = 0.94	Heterogeneity was inevitable	Development of targeted therapy	[70]
	FAM66C, CDKN2A-DT, AL359232.1, AL157888.1, FRMD6-AS1, AC023090.1, AC027237.4, AC026355.2, AL627443.3, FAM83A-AS1, AC008957.1, AP000346.1, GLIS2-AS1	LUAD	RT-PCR and cell culture	CRlncSig	-	Limited number of samples, Database updates	Early diagnosis and prediction of prognosis and treatment	[71]
circRNA	circNRIP1, circRANGAP1, circCORO1C, and circSHKBP1	GC	DNA and RNA sequences	MLP	-	A limited number of samples	Early diagnosis	[72]
snoRNA	HBII-52-14, SNORD123, HBII-336, HBII-85-29, U3, HBII-420, HBI-43, SNORA73B, SNORD116, SCARNA4, HBII-85-20	31 cancer types	-	MCFS, IFS, MCC, RIPPER	-	Sample size, in vivo, and in vitro studies are required	Expression pattern of snoRNAs in different cancers	[73]
	One hundred thirteen survival-associated snoRNAs were discovered.	HNSCC	Clinical databases	GSEA	AUCs = 0.674, 0.704, and 0.66 at 1/3/5 years, respectively.	First, the study primarily concentrated on data mining and analysis grounded in methodology without validating the results through experiments. Second, a limited database was obtained.	Early diagnosis and prediction of prognosis	[74]

Gholizadeh et al. attempted to identify mRNAs with the use of bioinformatics analysis and ML algorithms; they also created a screening technique that differentiates between healthy and HCC tissue. Together with three diagnostic biomarkers (AFP, CYP2E1, and ARK1C3), this work also yielded four extra prognostic markers (MAGEA6, RDH16, SOCS2, and RTN3) [75]. Further, Zhang and Liu used high-throughput omics data from the Cancer Genome Atlas (TCGA) in their investigation [76]. To choose six essential feature subsets that would function as reliable biomarkers, they concentrated on the ML feature selection technique and subsequently examined the efficacy of the biomarkers that had been discovered. They discovered that a number of significant genes, including EPHB1, SKAP1, STC2, CDHR2, MUC6, FAM134B, PHOSPHO1, and OXT, which are all connected in some manner to the onset of HCC, overlapped.

These biomarkers, which have been demonstrated to be strongly correlated with the initiation and spread of HCC, provide support for this methodology. Similarly, Zhao et al. identified differentially expressed mRNA and miRNA biomarkers for HCC detection using machine learning, particularly the random forest technique [63]. SFRP1, EDNRB, NR4A3, FHL2, NKX3-1, IL6ST, and FOXO1 are the mRNAs that are targeted by five miRNAs (hsa-miR-183-5p, hsa-miR-10b-5p, hsa-miR-224-5p, hsa-miR-10b-3p, and hsa-miR-182-5p) that have been linked to the development of HCC carcinogenesis in order to provide diagnostic and prognostic information provides a summary of these studies [63]. Beyond comparing normal tissue to HCC tissue, a meta-analysis of gene expression profiles discovered biomarkers to distinguish between HCC and cholangiocarcinoma through transcriptome networks [77].

A powerful cancer diagnostic tool built with an efficient machine learning approach is serum miRNAs with mutation-targeted RNA modification. An improved machine-learning approach was used by Liao et al. to investigate whether RMvar-related miRNAs were significantly Linked to carcinogenesis in order to identify oncological signatures formed from these miRNAs with variations targeting RNA modifications. With the help of 43,047 clinical samples and 504 serum RMvar-related miRNAs, nine distinct ML algorithms were used to create a diagnostic signature for detecting cancer (with an AUC value of 0.998, specificity of 93.1%, and sensitivity of 99.3% in the validation cohort) [78].

## The applications of RNA biomarkers for cancer management

### AI-enabled RNA-Based early cancer diagnosis

**Fig. 3:**
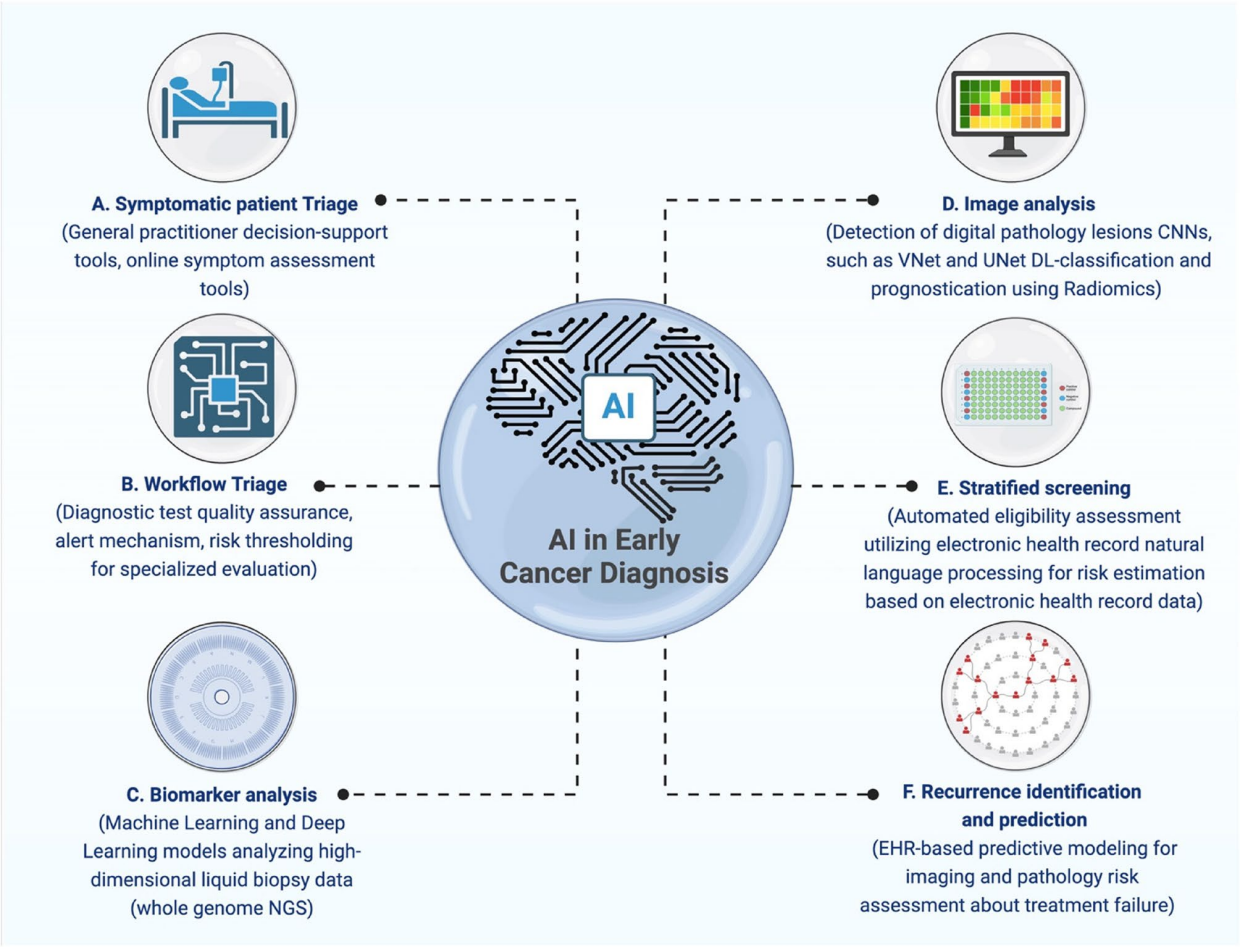
The schematic illustration represents the role of the utilization of AI algorithms for early cancer diagnosis. The illustration was created using BioRender.com

An important step forward in molecular biology has been the discovery of RNAs as potential biomarkers for the early identification of cancer (Fig. 3). One of the RNA biomarkers, such as specific miRNA signatures, may assume a substantial role in the early and accurate detection of tumors that are still not visible. For instance, Lu et al. [79] screened the miRNA expression profiles from over 300 patient-derived biosamples, including samples from various malignancies. MiRNA profiling alone could properly classify weakly differentiated cancers, unlike their extremely inaccurate mRNA counterparts. This showed the promise of miRNA profiling for cancer identification.

In addition, Shi et al. recently studied miRNAs as a potential tool for early pancreatic cancer (PC) diagnosis in patients with chronic pancreatitis [80]. The study effectively used a robust rank aggregation (RRA) machine learning technique to evaluate the expression profile of prospective miRNA biomarkers for early pancreatic detection. For example, miR-205-5p, found in serum, was found to distinguish between Pancreatitis and PC patients with an accuracy rate of 91.5%. Futhermore, the results showed that miR-205-5p expression could be used as a biomarker for advanced disease. Specifically, tumor specimens with high expression rates within the R1/2 resection margins (indicating residual tumor) had a worse prognosis compared to tumor specimens with low expression within the R0 resection margins. Applying evolutionary-driven AI learning approaches to cancer detection and early treatment decisions can greatly enhance outcomes, including overall survival rates and reductions in cancer recurrence [81].

The CancerSig program is an evolutionary learning example; it utilized a bi-objective combinatorial genetic algorithm to understand miRNA signatures that may facilitate the early identification of cancers. The expression for this is C (n, m), where ‘m’ is a miRNA profile that is specific to a cancer stage and ‘n’ is a pool of 7,117 possible miRNAs harvested from 4,667 patients with 15 distinct cancer types, including HCC, from TCGA. The pan-cancer miRNA signature study showed that three miRNAs (let-7i-3p, miR-362-3p, and miR-3651) could differentiate between tumor and non-tumor samples. Eight of the fifteen cancer types that were studied had these three miRNAs significantly impact stage prediction [82].

Possible diagnostic miRNA biomarkers for PC have been identified using alternative techniques that integrate machine learning and bioinformatics. The study examined three distinct datasets obtained from the Gene Expression Omnibus (GEO) database, each of which contained serum-derived miRNA expression profiles. Three ML algorithms, Random Forest, Least Absolute Shrinkage and Selection Operator regression analysis, and Support Vector Machine (SVM), found three candidate miRNAs (miR-125b-1-3p, miR-4648, and miR-3201) that showed potential as diagnostic biomarkers because of their changed differential expression patterns. With reported AUC values of 0.926 and 0.935, respectively, the combined model demonstrated remarkable performance and accuracy during training and validation [83].

### AI cancer stage prediction using RNA biomarkers

The assessment, which is based on the eighth edition of the TNM staging system developed by the American Joint Committee on Cancer, holds the greatest significance, just as it does in the case of the management of the majority of solid tumors. Since the cancer might vary greatly at different stages, this staging approach is essential for determining options for therapy and prognosis [84].

AI is revolutionizing many aspects of cancer research and treatment, particularly in areas such as diagnosis, prognosis, and therapy selection. A highly promising use of AI in oncology is a cancer-stage prediction based on RNA biomarkers. RNA biomarkers, including mRNA and non-coding RNA (miRNAs and lncRNAs), play crucial roles in gene expression and regulatory processes within cells. Dysregulation of these RNAs is often associated with cancer progression and can offer significant insights into the stage and aggressiveness of a tumor [85–87].

AI techniques, particularly ML and DL, are increasingly being used to analyze RNA expression profiles from cancer patients and predict the stage of the disease [88]. The expression levels of these RNA biomarkers vary according to cancer type and stage. As cancer progresses from early stages (localized) to advanced stages (invasive or metastatic), specific RNA biomarkers become upregulated or downregulated, providing clues about the tumor’s aggressiveness.

AI systems typically work with data generated from high-throughput RNA-seq or microarray technologies. These platforms can measure the expression levels of thousands of RNA molecules in a sample [89]. Preprocessing steps such as normalization, noise reduction, and feature selection are crucial. AI models need clean, high-quality data to detect relevant patterns. Feature selection techniques like Recursive Feature Elimination (RFE) or Principal Component Analysis (PCA) help in reducing the number of irrelevant or redundant RNA markers [90]. Moreover, AI models such as SVM, Random Forests, and Neural Networks are trained on labeled RNA biomarker datasets for cancer stage prediction, such as lymph node metastasis (LNM) and distant metastasis. Recently, DL techniques such as convolutional neural networks (CNNs) have been utilized to assist medical professionals in identifying lymph node metastases through ultrasound images [91]. Identifying LNM by ultrasonic imaging is a straightforward and precise diagnostic method; nevertheless, diagnosis frequently comes post-metastasis.

Numerous studies have documented atypical gene expression during lymph node metastases. The research conducted by Okugawa et al. showed that KiSS1 expression is significantly associated with LNM in colorectal cancer [92]. Zhang et al. forecasted LNM utilizing differentially expressed mRNAs and ncRNAs [93].

Expression data for mRNAs and ncRNAs serve as essential tools for the investigation and prediction of LNM. The progression and spread of cancer are more accurately elucidated by the dysregulation of gene connections than by isolated genes alone. For instance, AKT1 is aberrantly expressed in various cancer types, and its upregulation has been associated with LNM. However, new research has shown that in tongue squamous cell carcinoma, miR-138 regulates AKT1 expression through binding to AKT1 [94]. Conversely, miR-519d suppresses LNM by modulating MMP3 in oral squamous cell carcinoma and BC [95].

There are novel opportunities for understanding omics data in the area of cancer that have opened up to the studies of ceRNA networks in various types of cancer. Numerous computational techniques have been created to establish a ceRNA network for metastasis prediction. Lee et al. devised a novel approach for predicting LNM and distant metastasis by analyzing differential correlations between miRNAs and their target RNAs in cancer, utilizing extensive RNA-seq and clinical data. The differential correlations between miRNAs and their target RNAs demonstrated that miRNA-RNA correlations and networks comprising miRNA-RNA pairs were more effective in predicting prognosis and metastasis, utilizing SVM with a radial basis function (RBF) kernel and logistic regression (LR) machine learning algorithms [96].

Li et al. developed novel methodologies to extract significant gene characteristics and trained machine learning classifiers to predict stages of renal cell carcinoma samples. They used multivariate Cox regression with Elastic-net, Lasso, and Adaptive lasso penalties and best subset regression analysis to identify prognostic-associated miRNA signatures and predict early and late tumor stages of ccRCC using miRNA expression profiles. This approach is assessed based on RNA expression values of clear cell renal cell carcinoma obtained from TCGA [97]. Further, in order to improve the accuracy of bladder cancer staging, Qureshi et al. proposed the use of artificial intelligence and MRI/RNA-seq-based radio genomics. They suggested a model that distinguished between intra- and extra-bladder cancer with mean sensitivity, specificity, and accuracy of 94%, 88%, and 92%, respectively [98]. Similarly, Sathipati and Ying proposed an SVM-based classifier and SVM-BRC, to classify patients with BC into early and later stages. The researchers analyzed miRNA expression profiles in a cohort of 386 BC patients sourced from the TCGA. SVM-BRC identified 34 out of 503 miRNAs as a signature, with a 10-fold cross-validation mean accuracy of 80.38%, with sensitivity, specificity, and Matthews correlation coefficient values of 0.79, 0.81, and 0.60, respectively [99].

Overall, the accuracy and sensitivity of AI in relation to RNA-based biomarkers well-established tumor characteristics distinguish its stage, resulting in effective cancer management. However, there are still major obstacles in the way of standardizing tumor stage prediction and guaranteeing accurate results, both of which are essential for efficient cancer therapy.

### AI facilitates predicting cancer recurrence based on RNA biomarkers

Considering the long-term targets for treatment, it is beneficial to know which patients are more likely to experience a tumor recurrence. It will also assist physicians and patients in getting a clear view of how the disease progresses, allowing for more targeted treatment plans.

**Fig. 4:**
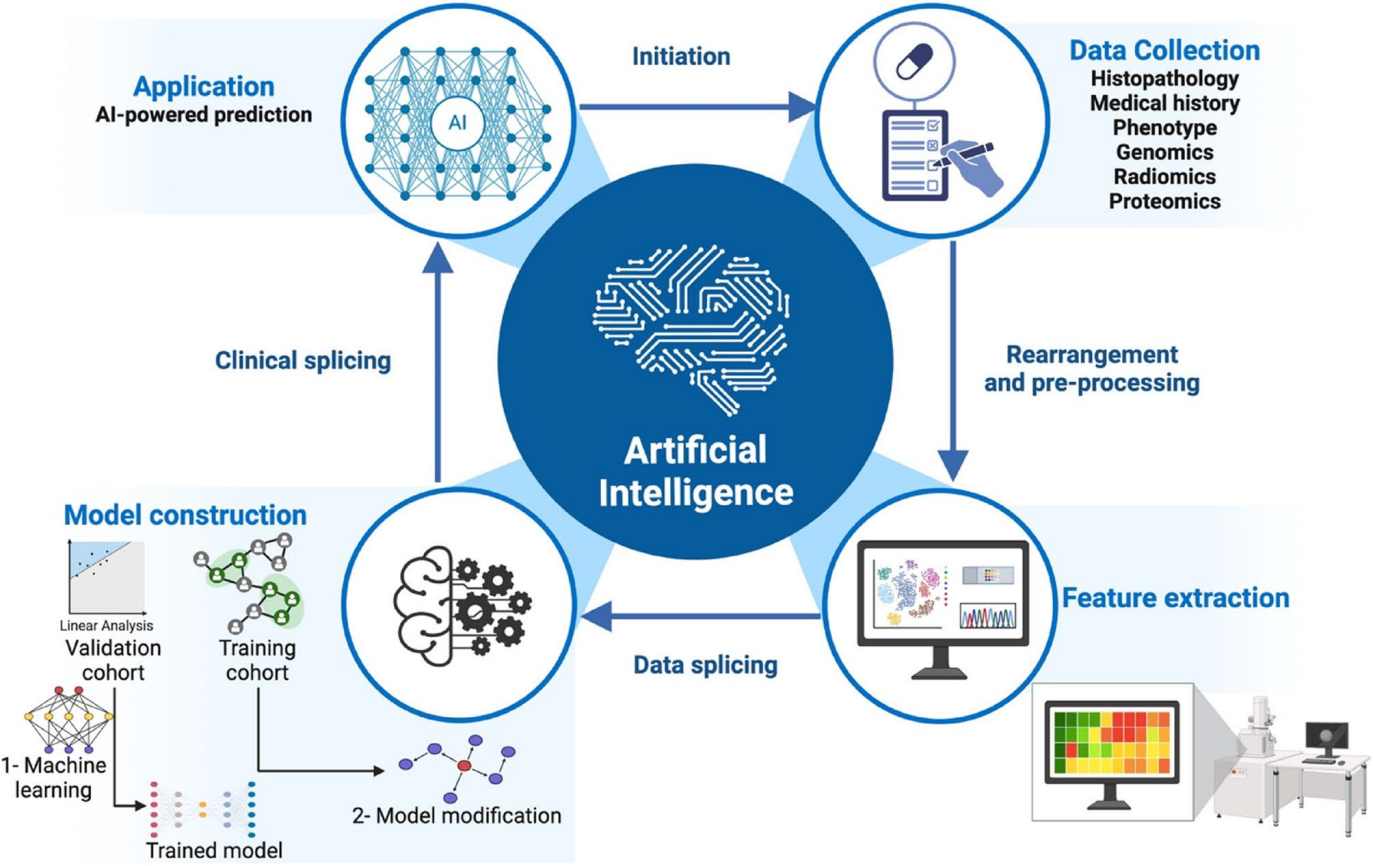
The schematic diagram represents the role of artificial intelligence in cancer diagnosis and therapy, as well as its current status and future perspective. The process starts with gathering data from various sources such as histopathology, medical history, phenotype, genomics, radiomics, and proteomics. Next, the data is adjusted and prepared, important features are identified, the data is divided into training and validation groups, a model is built using machine learning methods and refined, clinical data is used for validation, and finally, AI is applied for predictions in healthcare. Created with BioRender.com

AI can significantly influence clinical results and treatment plans by improving the accuracy of tumor recurrence predictions (Fig. 4). With a discriminatory power of 89.5%, Rodriguez-Luna et al. used an ANN with microsatellite mutation or deletion genotyping to predict HCC tumor recurrence in 19 patients who had liver transplants [100]. Using ML and public databases, Shen et al. formulated a prediction model for the recurrence of HCC tumors. They validated the model by looking for genetic signatures that could be linked to recurrence [101]. Their model achieved a precision of 74.19% in predicting tumor recurrence by combining AI with conventional statistical approaches like chi-squared tests and survival analysis.

On the same basis, Fu et al. created an AI model for the early detection of HCC recurrence prediction using ncRNA, such as lncRNA characteristics acquired from machine learning in conjunction with TNM stages and AFP values [102]. They used multivariate Cox analysis and three ML algorithms-LASSO, random forest, and SVM-RFE to choose the proper lncRNA signatures. According to their multivariate analysis, there were three independent indications of HCC recurrence: the ML risk score (HR = 1.5, *p* = 0.015), AFP levels (HR = 1.74, *p* = 0.012), and TNM staging (HR = 2.01, *p* = 0.01 for stage III + IV).

Research in this area has additionally attempted to predict cancer recurrence by creating and validating many non-coding gene signatures using AI and ML approaches [103, 104]. For example, the HSIC algorithm identifies the association between the recurrence of non-coding gene expressions and the recurrence of cancer genes controlled by several risk factors. Srividhya et al. used the HSIC model with a correlation matrix to find lncRNAs that may predict which cervical cancer (CC) genes would become recurrent. They set up the recurrent RNN model to find the hub genes associated with CC recurrence. They could predict the spread of CC to some degree using an LSTM model. Their study used the Artificial Fish Swarm Algorithm (AFSA) to identify CC cells that proliferate. They suggested a new approach that solves the issue of risk value using positive and negative scores that indicate a high chance of CC recurrence and a low risk of CC, respectively. The proposed method reveals a positive risk score of 45.987 and a negative risk score of −32.654 [105].

## Challenges of applying AI-based models for RNA biomarker analysis in cancer

**Fig. 5:**
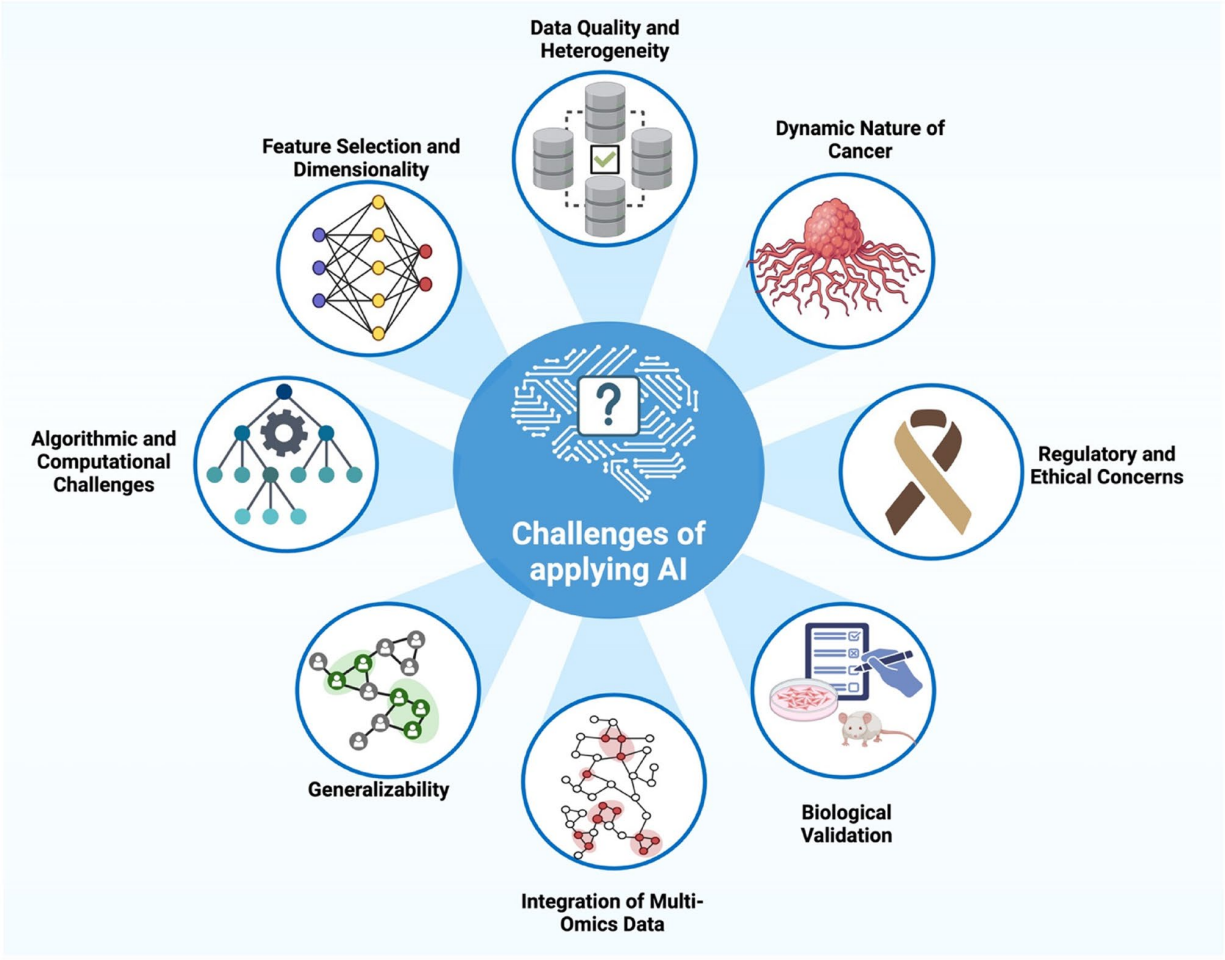
Using AI in cancer research and treatment presents several important obstacles. Data quality and heterogeneity issues, the ever-changing cancer biology, challenges with feature selection and high-dimensional data analysis, computational and regulatory limitations, ethical considerations, biological validation needs, integration of multi-omics datasets, and generalizability across different patient populations and clinical settings are the eight main obstacles shown in the diagram. The diagram was created using BioRender.com

Despite the potential benefits of AI-based models, these methods are limited when applied to RNA biomarker analysis in cancer research (Fig. 5). One such barrier is the lack of large-scale, open-source datasets that have been phenotypically characterized; this is often due to expensive processing costs and limited sample availability. In addition, tumor samples are usually kept in formalin-fixed paraffin-embedded (FFPE) blocks, which damages the RNA and makes them unusable for sequencing, profiling, and data generation [106, 107]. The fact that most AI systems produce point-estimate predictive methods raises concerns about the potential for confidence in predictions and incorrect diagnoses when used in clinical settings. One possible solution could be to use a Bayesian method, like the one described in the new paper on Epistemic Invariance in Cancer Classification (EpICC) [108]. In addition, there is a lot of noise and bias in RNA sequencing data since different platforms, methods of sample collecting, and processing all contribute to the data mismatch [109]. The solution maintains uniformity by executing stringent preprocessing procedures, including quality filtering, normalization, and batch effect correction [110].

Another issue is the lack of comprehensive annotated RNA databases, especially for rare cancers, where massive datasets are hard to discover. Collaborative, multi-institutional data-sharing networks can help overcome the lack of complete annotated RNA databases [111, 112]. The statistical strength and depth of annotations can be enhanced by merging data from several research centers and hospitals to build a more diverse and vast collection of RNA samples. Synthetic data creation techniques, like AI-based predictive modeling, can also be helpful in situations where there is a lack of actual data to recreate RNA expression patterns [113].

Still a significant issue, the experiments of Nirmal et al. [114] and the data acquired by Frasca et al. [115] highlight the need to combine ML with domain-specific knowledge. Effective collaboration between data researchers and domain specialists is the only way to construct models, interpret results, and align algorithms with domain-specific requirements and restrictions.

According to the EU’s AI Act, which emphasizes the need for algorithms to provide “sufficient transparency” for users, the explainability of AI systems is now mandated by law for high-risk applications [115]. The subject matter is crucial because it involves understanding model operational dynamics and delivering valuable insights to non-experts to ensure responsible, ethical, and defensible AI use.

Despite these challenges, due to their profound clinical implications, there remains considerable enthusiasm for leveraging computational tools to efficiently and accurately predict the presence and stage of malignancies, including previously undetectable tumors. To conclude, future translational perspectives suggest that integrating AI-based models with high-quality, standardized RNA datasets and explainable frameworks, alongside collaborative multi-institutional networks, could drive the clinical application of RNA biomarkers toward precision oncology and intelligent medicine.

## Discussion

The integration of AI with RNA biomarker research is reshaping the landscape of cancer diagnostics and therapeutics [116]. The reviewed literature underscores AI’s capacity to enhance the discovery, validation, and clinical application of diverse RNA molecules, including miRNAs, lncRNAs, and circRNAs [117]. These non-coding transcripts, once considered transcriptional byproducts, have now been established as critical regulators of oncogenic and tumor-suppressive pathways. By coupling high-throughput transcriptomic technologies with AI algorithms, researchers can extract clinically relevant patterns from complex datasets that conventional analytical methods often fail to resolve [118].

One of the most significant findings highlighted in this study is AI’s contribution to early cancer detection and staging. Models such as support vector machines, random forests, and deep neural networks have demonstrated high accuracy in distinguishing malignant from benign tissues, classifying tumor subtypes, and predicting recurrence risks [119]. For example, the use of miRNA expression profiles coupled with AI has been shown to outperform mRNA-based approaches in the early classification of poorly differentiated tumors [120]. Similarly, the identification of lncRNA signatures predictive of survival or recurrence illustrates how AI enables a more granular stratification of patients, advancing the goals of precision oncology [121].

Despite these promising outcomes, several limitations temper the clinical translation of AI-assisted RNA biomarker analysis. A persistent challenge is the lack of sufficiently large, standardized, and phenotypically annotated datasets, particularly for rare cancers [122]. Differences in sample handling, sequencing platforms, and computational pipelines introduce variability that can bias model training and compromise reproducibility [123]. Another obstacle to clinical use of AI is the difficulty in understanding and using complicated models, even if AI is great at pattern detection [124]. Clinicians require transparent and biologically meaningful explanations for algorithmic predictions to build confidence and ensure regulatory compliance, as emphasized by frameworks such as the EU AI Act [125].

The ethical and practical implications of integrating AI into oncology must also be carefully addressed. Issues such as data privacy, potential biases in training datasets, and equitable access to AI-driven technologies are critical considerations for responsible implementation [126]. Collaborative, multi-institutional data-sharing initiatives and understandable AI models represent viable strategies for overcoming these barriers [127]. Furthermore, hybrid approaches that combine AI-driven predictions with established clinical parameters, such as TNM staging and biomarker panels, may yield more robust and clinically acceptable outcomes [116].

Looking forward, the convergence of AI, RNA biology, and multi-omics integration offers a path toward more intelligent cancer care. AI-driven models can facilitate not only the early detection and stratification of patients but also the identification of novel therapeutic targets and the optimization of RNA-based therapies, such as siRNAs and antisense oligonucleotides. To make this ambition happen, though, clinicians, statisticians, molecular biologists, and government agencies will need to work together. By bridging these domains, AI-enabled RNA biomarker research can transition from proof-of-concept studies to routine clinical practice, ultimately improving patient outcomes and reducing the burden of cancer care.

## Conclusion

This comprehensive study demonstrates how the combination of AI and RNA biomarker analysis has produced notable improvements in cancer prognostics and diagnosis. AI-based techniques showed a mean diagnostic accuracy of roughly 90–92% throughout the reviewed research, with ensemble and DL approaches often reaching > 95% accuracy in certain datasets. Sensitivity values averaged around 92–94%, while specificity was generally maintained at 88–90%, indicating robust classification capabilities. Significantly, the reported AUC values were consistently high, averaging 0.90–0.95, with some ML models such as Random Forest, XGBoost, and Artificial Neural Networks reaching near-perfect performance (AUC ~ 1.0).

When these findings are combined, the average accuracy of ~ 91%, sensitivity of ~ 93%, specificity of ~ 89%, and AUC of ~ 0.92 across several datasets can be used to describe the overall performance of AI-RNA biomarker models. These benchmarks indicate the consistency and dependability of AI-driven methods in cancer analysis based on biomarkers.

ML techniques, such as logistic regression, decision trees, random forests, and gradient boosting, have attained 91–98% accuracy, with specificities close to 100% and sensitivities frequently surpassing 95%. Similar to classical models, DL frameworks like hybrid architectures and multilayer perceptrons showed better performance. Despite producing lower accuracies (~ 73%), feature selection techniques offered crucial dimensionality reduction that improves interpretability and computational effectiveness. With AUCs ranging from 0.74 to 0.98 over 1-, 3-, and 5-year survival intervals, models including RSF, CoxBoost, and RDeepNet demonstrated clinically relevant predictive power in survival prediction.

All of these findings point to AI-driven models as effective instruments for cancer analysis based on RNA biomarkers. The data highlights their ability to find clinically useful biomarkers with significant translational potential in addition to effectively classifying tumor subtypes. To fully achieve AI’s potential in precision oncology, however, the research also highlights important obstacles that need to be overcome, such as data heterogeneity, interpretability issues, and a lack of clinical validation. With the goal of enhancing diagnostic precision, treatment choices, and patient outcomes, this study offers a roadmap for the clinical application of AI-enabled RNA biomarker analysis in the future by combining performance metrics and emphasizing the best algorithm-biomarker combinations.

## Data Availability

No datasets were generated or analysed during the current study.

## Abbreviations

ACA: Adrenocortical carcinoma and adenoma
AI: Artificial intelligence
BC: Breast cancer
CRC: Colorectal cancer
EpICC: Epistemic Invariance in Cancer Classification
GC: Gastric cancer
HCC: Hepatocellular carcinoma
HNSCC: Head and neck cancer
LUAD: Lung adenocarcinoma
ML: Machine learning
OC: Ovarian cancer
STAD: Stomach adenocarcinoma
SVM: Support Vector Machines
